# Supplementary material for: Causal Relationship between Aging and Anorexia Nervosa: A White-Matter-Microstructure-Mediated Mendelian Randomization Analysis
Source: Biomedicines. 2024 Aug 16;12(8):1874. doi: 10.3390/biomedicines12081874 (PMC11351342; doi:10.3390/biomedicines12081874)
Supplement: Supplementary file 1 [file biomedicines-12-01874-s001.zip › Supplementary Figure/Figure S9/Leaveoneout plot of MR analysis for ubm-b-1871 to AN.pdf]

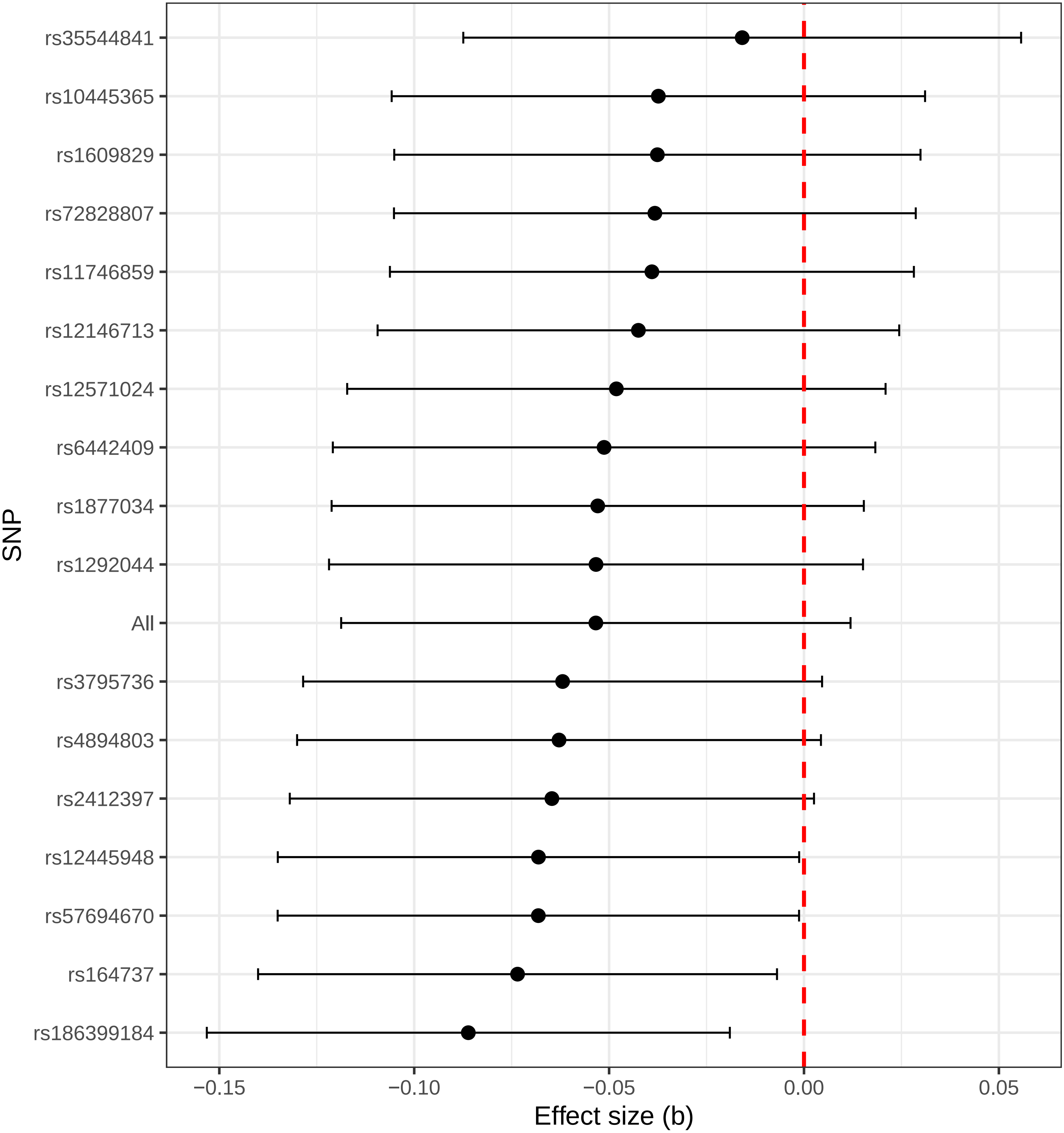
rs35544841
rs10445365
rs1609829
rs72828807
rs11746859
rs12146713
rs12571024
rs6442409
rs1877034
rs1292044
All
rs3795736
rs4894803
rs2412397
rs12445948
rs57694670
rs164737
rs186399184
SNP
−0.15
−0.10
−0.05
0.00
0.05
Effect size (b)
